# Cross-sectional study of the prevalence of hyperextension of the first metatarsophalangeal joint and its relationship to onycholysis in women with hallux valgus

**DOI:** 10.1186/s12891-024-07219-1

**Published:** 2024-02-05

**Authors:** Rosana Giménez-López, Carlos Barrios-Pitarque

**Affiliations:** 1 School of Doctorate, Catholic University of Valencia “Saint Vincent Martyr”, Plaza de San Agustín, 3 Esc. A, Entresuelo, 46001 Valencia, Spain; 2 Institute for Research On Musculoskeletal Disorders, Catholic University of Valencia “Saint Vincent Martyr”, Quevedo 2, 46001 Valencia, Spain

**Keywords:** First Metatarsophalangeal joint, Hyperextension, Hallux valgus, Onycholysis

## Abstract

**Background:**

Hallux Valgus (HV) deformity is associated with misalignment in the sagittal plane that affects the first toe. However, the repercussions of the first toe hyperextension in HV have been scarcely considered. The purpose of this study was to provide evidence of the association between first-toe hyperextension and the risk of first toenail onycholysis in HV.

**Methods:**

A total of 248 HV from 129 females were included. The extension of 1st MTP joint was measured while the patient was in the neutral position of the hallux using a two-branch goniometer. The classification of the HV severity stage was determined by the Manchester visual scale, and the height of the first toe in the standing position was measured using a digital meter. An interview and clinical examination were performed to collect information on the presence of onycholysis of the first toe.

**Results:**

Of the 248 HV studied, 100 (40.3%) had onycholysis. A neutral extension > 30 degrees was noted in 110 (44.3%) HV. The incidence of onycholysis was higher in HV type C than in type B (*p* = 0.044). The probability of suffering onycholysis in the right foot was 2.3 times greater when the neutral position was higher than 30 degrees (OR = 2.3; *p* = 0.004). However, this was not observed in the left foot (*p* = 0.171). Onycholysis was more frequent in HV with more than 2 cm height of the first toe (*p* < 0.001). For both feet, the probability of suffering onycholysis was greater for each unit increase in hallux height (right foot OR = 9.0402, *p* = 0.005; left foot OR = 7.6633, *p* = 0.010).

**Conclusions:**

The incidence of onycholysis appears to be significantly associated with HV showing more than 30º extension, and more than 2 cm height of the first toe. Height and hyperextension of the first toe together with first toenail pathology should be mandatory in the evaluation of HV.

## Background

Hallux valgus (HV) is a complex three-dimensional deformity usually associated with hyperextension of the hallux. Predisposing factors for HV include hereditary aspects, biomechanical compensation of the lower limb, metatarsus adductus, flat foot, foot morphology, gastrocnemius shortening, narrow shoes, and joint hyperlaxity [1–3]. The bifurcation or accessory tendon of the extensor hallux longus (EHL) has also been reported to be a predisposing factor for HV [4, 5].

HV is one of the most common pathologies of the forefoot. Its prevalence is estimated to be 23% in adults between 18 and 65 years of age and 35.7% in those older than 65 years. This percentage increases with age, and women are usually more affected than men are, with a ratio of 15:1 [6–8]. With age, progressive joint deterioration and subsequent mobility limitations of the first metatarsophalangeal (1st MTP) joint are observed [9, 10].

The muscle imbalance resulting from deviation in the HV is evident. The EHL and extensor hallux brevis (EHB) tendons play a relevant role in hallux hyperextension in all degrees of HV deformity due to medial deviation of the first radius and lateral deviation of the first toe. The EHL exerts a bowstring-like tension that facilitates hyperextension and sometimes supraduction of the first toe over the second toe. At the same time, the flexor hallux longus (FHL) tendon rotates laterally and pulls the distal phalanx into the valgus position. The function of these muscles is impaired due to deformity, promoting instability of the first metatarsophalangeal segment during gait [11, 12].

Among the most common alterations due to the lack of correct support of the hallux are nail disorders, such as onychocryptosis [13]. The literature also refers to cases of distal phalanx hyperextension in hallux limitus pathology, which frequently suffer from trauma to the distal part of the toe against the shoe’s upper area, possibly leading to the formation of subungual exostoses [14].

Primary or idiopathic onycholysis (IO) is described as the distal or distolateral separation of the nail plate from the nail bed, and/or the underlying lateral supporting structures (hyponychium, lateral nail folds). The nail acquired a whitish colouration due to air entrapment and may contain keratinous debris. Its histopathology is nonspecific and frequently observed in adult women [15, 16]. Generally, it is the precursor lesion of onychomycosis and distal subungual hyperkeratosis, and it is usually asymptomatic. Some authors note that many negative fungal cultures, or even if they are positive, do not indicate clinical symptoms [16–19]. In studies involving 3,000 abnormal toenails, only approximately 30% were culture positive for dermatophytes [20]. If onycholysis persists, it can cause the disappearance of the nail bed (DNB). The nail bed keratinises and produces dermatoglyphs like a normal fingerprint. In toenails, contributing factors include increasing age, history of trauma, surgery, onychomycosis and onychogryphosis. [16, 21]. The exact incidence of both onycholysis and DNB is unknown. Both lesions are easily recognized at a clinic by an experienced professional [22]. In addition, a group of clinical abnormalities, including onycholysis, nail bed keratosis, and other nail plate abnormalities, have been called “AGNUS” (asymmetric gait nail syndrome). It has been reported as the most common toenail abnormality worldwide in shoe-wearing societies. It is generally produced by a repetitive mechanical stress, specifically due to hallux rubbing against footwear [23, 24].

It is important to point out that, in HV deformity, the misalignment in the sagittal plane mainly affects the first toe. This alteration has been usually considered less relevant, but it is certainly important given the toenail repercussions that this alteration may cause.

To our knowledge, there are scarce references in the scientific literature regarding the possible relationships between hallux hyperextension and nail disorders. Therefore, the main objective of the current study was to assess the presence of hallux hyperextension in neutral position considering the different stages of severity of the deformity and its possible association with onycholysis. A second objective was to provide information on the clinical characteristics of onycholysis in females with HV and the possible relationship between the height of the first toe and the ungueal lesion.

## Methods

### Study design

A cross-sectional, observational, analytical study was conducted. A non-probabilistic and consecutive sampling was performed in a podiatric clinic located in Valencia (Spain) from January 2021 to February 2022. The sample was taken from women who attended consultation for different foot problems. Prior to their clinical examination, all patients signed an informed consent. Ethical approval was obtained from the Research Ethics Committee of the Catholic University of Valencia (CEI) on January, 2021. Project code: UCV/2020–2021/052.

Sample calculation was performed to estimate the proportion of patients with HVs who presented nail disorders at the first nail. A minimum sample size of 93 patients was required considering a confidence level of 95% and a precision of ± 10% of cases, estimating that this proportion would be approximately 60%.

Inclusion criteria were as follows: aged between 20 and 70 years with HV, regardless of the severity of the deformity. Exclusion criteria included: patients having metabolic, neurological, rheumatological or vascular disease; previous locomotor diseases; previous surgery; supra- or infraductus hallux, and hallux limitus (passive extension of the 1st MTP joint less than 65 degrees) [25]. In relation to nail pathology, there were excluded patients with a previous history of direct nail trauma, and positive and no valid tests for dermatophytes.

### Assessment of hallux hyperextension and severity of the deformity

**Fig. 1:**
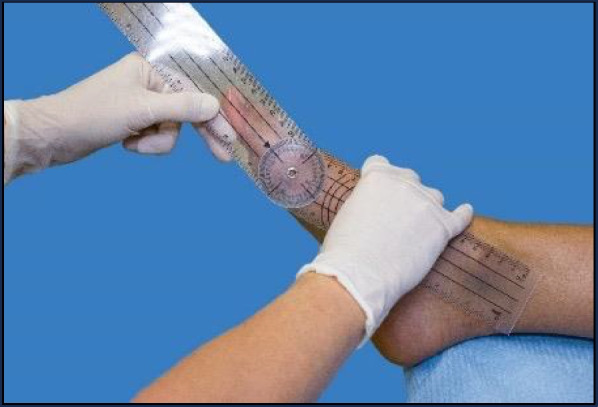
Extension neutral position measurement by goniometer

The 1st MTP joint was assessed in a neutral extension position while the participants were seated and with the subtalar joint in a neutral position. A two-branch universal goniometer was used for the measurements. The branches of the goniometer were placed following the previously marked bisectors of the first metatarsal and the proximal phalanx on the foot medial side (Fig. 1). The values were taken without any movement of the joint in the relaxed position. Measurements were taken on three consecutive occasions alternating both feet for average calculation. Twenty degrees was taken as a reference in the neutral position of the 1st MTP joint without pathology [26–29], considering a normal range up to 30°.

**Fig. 2:**
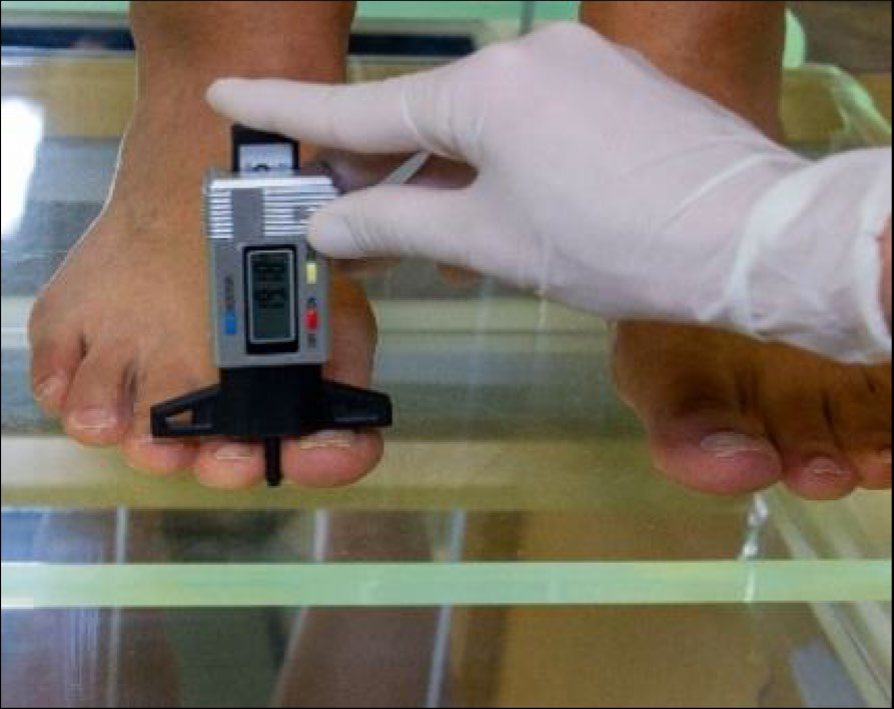
Height measurement by a digital profile depth gauge

Measurement of the height of the first toe in the standing position was assessed using a digital profile depth gauge, an industrial tool widely used in different areas (automation and others). The digital gauge was previously calibrated at 0 and placed in the first interdigital space by contacting one of its branches with the nail plate without pressure. Height was determined in cm (Fig. 2). All the measurements were taken consecutively three times, alternating between both feet.

The HV severity stage was established using the Manchester visual scale [30]. An inspection, analysis and evaluation questionnaire were administered to the nail appearance of the patients by a single observer with 22 years of clinical experience. The diagnosis of onycholysis was based on the patient’s clinical history and by performing the Diafactory® test, a rapid immunochromatographic test designed to search for dermatophyte antigens in nail samples. These antibodies strongly react with seven Dermatophytes (T. rubrum, T. mentagrophytes, T. violaceum, T. tonsurans, M. gypseum, M. canis and E. floccosum) [31].

### Statistical analysis

The statistical data analysis was carried out using R software (version 4.2.0). First, a descriptive analysis of the study variables was performed to determine demographic and clinical characteristics of the sample. Quantitative variables are presented as the mean, standard deviation, median, minimum, and maximum. Categorical variables are described by absolute and relative frequencies. To assess the relationship between HV severity (B, C, or D) and hallux hyperextension, a multinomial linear regression model was fitted. A linear regression model was used to test whether onycholysis was dependent on the Manchester grade, the degree of hyperextension (> or ≤ 30 degrees), and the hallux height. A 95% confidence level was used in all the patients.

## Results

### Patients profile

**Fig. 3:**
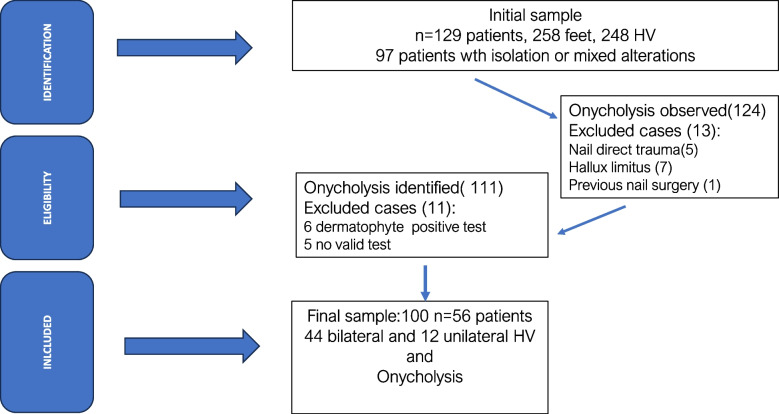
Sample selection process

Figure 3 show the flow chart of the patient’s identification, eligibility and final sample included in the study. The sample consisted of a total of 129 women with a mean age of 51 ± 11 years. Table 1 summarized the hallux valgus distribution according to foot side and the Manchester scale. The sample included 248 feet with HV. HV was observed bilaterally in 119 patients (92.2%), and unilaterally in 10 patients (7.8%).

**Table 1:** Demographic and clinical characteristics of patients

***Sample characteristics***	
	Median ± SD
Age	51 ± 11
Weight	66 ± 13
Height	162 ± 5
BMI	25.1 ± 4.7
**Hallux valgus** (*n* = 248)	N (%)
**Right side**	122 (49.1%)
Mild (B)	66 (26.6%)
Moderate (C)	50 (20.1%)
Severe (D)	6 (2.41%)
**Left side**	126 (50.8%)
Mild (B)	58 (23.3%)
Moderate (C)	57 (22.9%)
Severe (D)	11 (4.4%)

### Hallux hyperextension and HV severity

Table 2 show the first toe extension degrees in a neutral position for each foot side according to HV Manchester classification. Taking 20º as a reference of normality, a mean increase of 12 degrees in stage B, 9 degrees in C, and 6 degrees in D was observed in the neutral position in hallux hyperextension of the left foot. In the right foot, an increase of 12, 10, and 7 degrees were observed (Table 2). No significant association was found with any stage of HV according to multinomial logistic regression models for any foot (*p* > 0.05). Out of the 248 HV, 110 (44.3%) had an extension > 30 degrees in relaxed position.

**Table 2:** Sagittal extension degrees in neutral position in the diverse HV categories

	**HV severity (Manchester scale)**			
Side	**B** (mean ± SD)	**C** (mean ± SD)	**D** (mean ± SD)	Whole sample (mean ± SD)
Right Foot	32.30 ± 9.54	29.90 ± 9.57	26.66 ± 7.33	31.15 ± 9.52
Left Foot	32.14 ± 8.35	29.30 ± 9.39	26.39 ± 10.0	30.38 ± 9.07

*SD* Standard deviation

### Onycholysis

Among the 129 participants, 97 (75.1%) had several nail diseases in isolation or mixed. Among the multiple alterations found, 124 cases of HV with onycholysis were observed. Of these cases, 7 cases had hallux limitus, 5 had long-standing onycholysis due to direct trauma and 1 had previous nail surgery and 1 had vascular disease: all of whom were excluded. In the remaining 111 cases, 60 participants (51 with bilateral lesions and 9 with unilateral lesions), the Diafactory test for dermatophyte fungi was performed. The results showed 6 positive cases and 5 with no valid test: these cases were excluded. The final sample consisted of 100 negative dermatophyte onycholyses affecting 56 patients (43.4%) of the participants. There were bilateral lesions in 44 cases (78.5%) of patients, and unilateral lesions in 12 (21.4%).

With respect to the medical history of the 56 patients with onycholysis, the nail lesion was attributed to continuous nail microtrauma against the shoe box in 18 cases (32.1%), sometimes associated with nail pain. Other 13 patients (23.2%) had partial or splinter subungual hematomas. A group of 9 patients (16.1%) had received different treatments for onychomycosis without achieving any improvement for at least one year.

### Onycholysis and HV severity

**Fig. 4:**
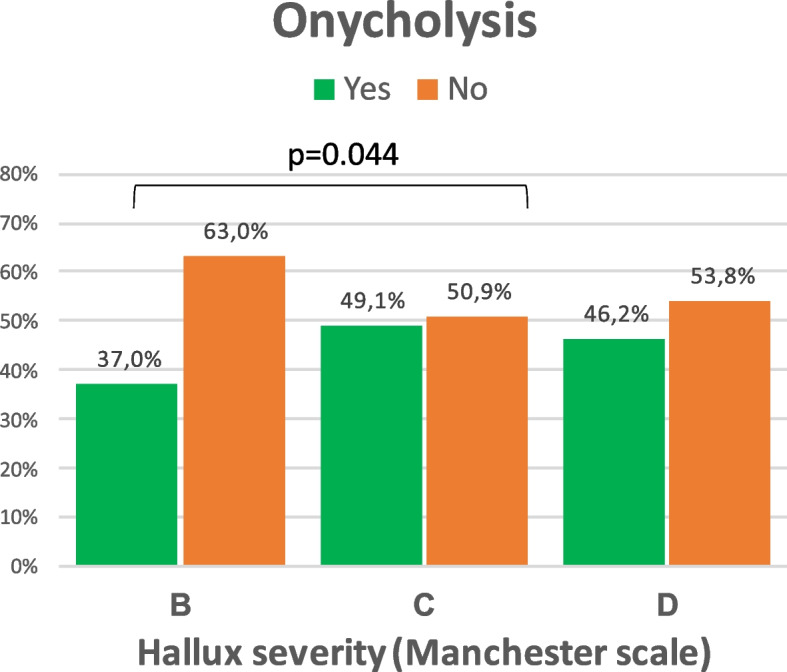
Incidence of onycholysis HV subtypre

Figure 4 shows the incidence of onycholysis in the three HV subtypes according to Manchester scale. Of the 124 HV Manchester subtype B, only 36.2% (47 cases) developed onycholysis. However, of the 107 HV Subtype C, 49.5% (53 cases) had onycholysis. Differences between these two subgroups were statistically significant (*p* = 0.044).

### Onycholysis and HV hyperextension

**Fig. 5:**
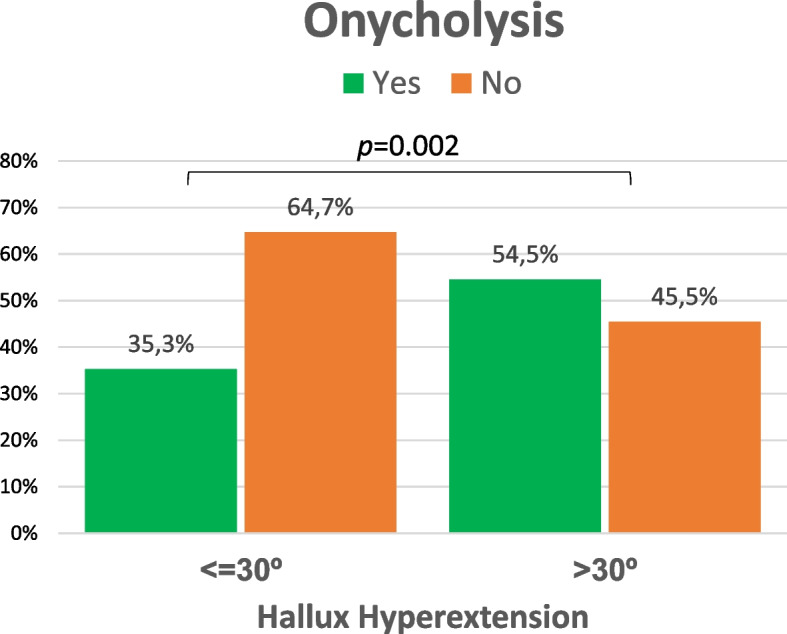
Incidence of onycholysis according to extension of the first toe

**Fig. 6:**
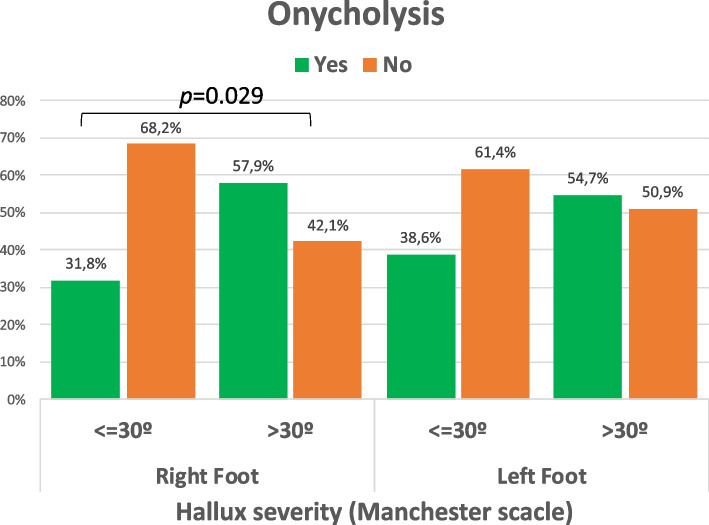
Onycholysis cases regarding side and extension degrees

According to the categorization of > or ≤ 30º of extension of the first toe in a neutral position, only 35.3% of the patients with ≤ 30º HV hyperextension showed onycholysis (48/136) (Fig. 5). In cases with > 30º HV hyperextension, the incidence of onycholysis was statistically significant higher (54.5%: 53/106: *p* = 0.002). When the two feet were analysed separately, the differences between the two groups were particularly increased in the right foot (Fig. 6).

HV with onycholysis showed a greater first toe extension than those without onycholysis, and the difference was statistically significant (*p* = 0.019). The median value for both first toe extension with onycholysis was 32.4º (Table 3). In the categorization > or ≤ 30 degrees of extension in a neutral position in relation to onycholysis, we observed that in the right foot, there was a high proportion of patients with extension > 30 degrees, 31/51 = 60.78% while in the left foot the ratio is lower(> 30 degrees 29/51 = 56.86%). It was observed that having an extension > 30 degrees means a 2.3 times increase in the probability of having onycholysis compared to not presenting it (OR = 2.298, *p* = 0.026). However, it was not found in the left foot (OR = 1.718, *p* = 0.916).

**Table 3:** First toe extension degrees and height in the presence or absence of onycholysis

	**Onycholysis** *n* = 100	**Nails without Onycholysis** *n* = 140	**F (*p*)**
**First toe extension**			
Mean ± SD (mm)	32.42 ± 9.58	29.66 ± 8.73	5.546 (*p* = 0.019)
95% CI	30.59–34.25	28.19–31.11	
Minimum–maximum (mm)	10–51	10–51	
**First toe height**			
Mean ± SD (mm)	2.16 ± 0.25	2.04 ± 0.25	13.290 (*p* < 0.001)
95% CI	2.11–2.21	2.00–2.09	
Minimum–maximum (mm)	1.30–3.00	1.50–3.00	

*SD* Standard deviation

### Onycholysis and height of the first toe

**Fig. 7:**
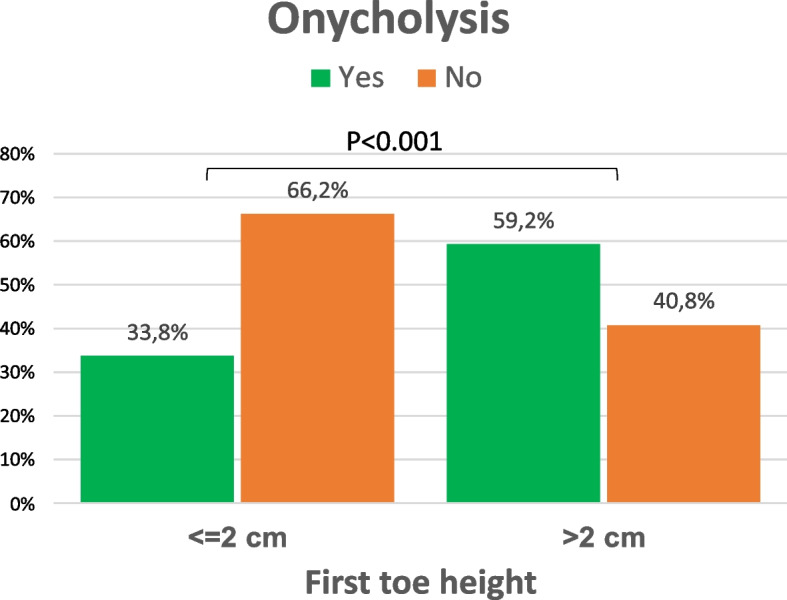
Onycholysis incidence according to the height of the first toe

HV with onycholysis showed a greater first toe height than those without onycholysis, and the difference was statistically significant (*p* < 0.001) (Table 3).According to the categorization of > or ≤ 2 cm of height of the first toe, only 33.8% of the patients with ≤ 2 cm showed onycholysis. In cases with > 2 cm of first toe height, the incidence of onycholysis was statistically significant higher (59.2%;* p* < 0.001) (Fig. 7).

**Fig. 8:**
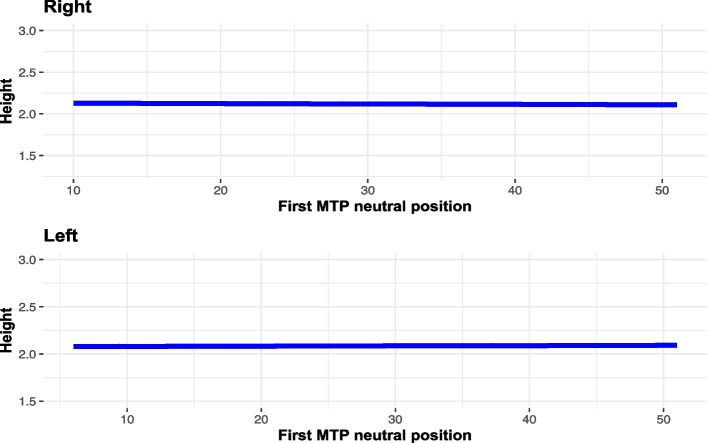
Correlattions between extension and height of the first toe

The average height of the first toe in standing position was 2.11 cm for the right foot, and 2.08 cm for the left foot. In both feet, the probability of experiencing onycholysis was greater for each unit increase in height (right foot: OR = 9.0402, *p* = 0.005; left foot: OR = 7.6633, *p* = 0.010) The 1st MTP joint extension in a neutral position was not related to the hallux height (*p* > 0.05) (Fig. 8).

## Discussion

This is the first study to investigate the extension degree in the neutral position of the first toe in the different severity stages of HV and its relationship with onycholysis. Clinical tests were performed to determine whether the possible cause of this nail lesion was related to hyperextension of the first toe in HV patients.

We could argue that the extensor hallux (EHL), due to its dorsal and lateral positioning in HV deviation, is the most important triggering factor of hallux hyperextension. However, tendons of the FHL and adductor muscles also play a triggering role in this deformity [12]. Yokozuka et al. [11] reported how HV reduces toe flexor strength. In addition, other authors have noted that a prolonged toe extension position can cause shortening of the extensor digitorum longus (EDL) muscle and lengthen the intrinsic toe muscles in hammer toe deformity; even the EDL could also contract in a more dominant manner than the tibialis anterior muscle [32].

We found that extension in HV patients decreases progressively by approximately 3 degrees for each increase in severity. Hence, hyperextension is inversely proportional to the severity of the deformity. It seems that onycholysis is not related to the severity of HV but rather to the hyperextension degree of the hallux, equal to or greater than 30 degrees, which constituted 83% of the cases in the whole sample.

In this study, we observed a high rate of pathology at the nail plate and nail bed, but onycholysis was found in a high percentage of the sample. According to previous studies, onycholysis is highly frequent and is caused by hallux rubbing against the shoe box [19, 22, 23]. It is also a pathologic condition that may precede onychomycosis, an infection that seems to not be as frequent as expected [24].

In the present study, a correlation was found between the height of the first toe on both feet in the standing position and onycholysis. We also analysed the possible relationship between extension and height of the hallux, but no association was observed. Hence, we hypothesize that the height of the first toe may be due to the dorsiflexed position of the distal phalanx or to the morphology of the toe.

The first toe is the largest toe and therefore the first to be rubbed by the top or distal part of the shoe in the absence of deformity of the lesser toes. The first protective barrier is the nail plate and, in patients with VH, the first toe, due to its elevation, will receive continuous microtrauma that will promote onycholysis. This rubbing will normally be asymptomatic. Add to this mechanism an ill-fitting shoe, either in length or width to accommodate the toes, will be another contributing factor to cause certain injury to the nail unit.

We have observed that the highest percentage of onycholytic lesions appears on the external or peroneal side of the first toe. This location may be the result of hallux pronation due to the biomechanics of this pathology, leaving the peroneal side higher and therefore more exposed to friction with footwear.

One limitation of this study was that the diagnosis of infectious agents such as bacteria or other fungi could not be ruled out. Fulfilling the exclusion criteria also favored a reduction in the sample of patients with a greater degree of severity of HV. Although footwear plays a determining role in nail pathology, especially in onycholysis, it was difficult to evaluate the average height of the shoe vamp due to the deformity of the vamp, the variety of footwear and the profile of the soles. The dynamic behaviour of the MTP joint within the shoe in cases of HV has also not been evaluated.

Hyperextension treatment in patients with HV involves specific surgical techniques, such as lengthening or reinserting the EHL tendon [33, 34]. A more conservative approach was considered by Baran and Badillet [18], who suggested a solution to avoid nail microtraumatism against the shoe. Sano et al. [15] also proposed a treatment to lower the toe and increase its bearing surface area in the case of distal phalanx dorsiflexion.

## Conclusions

It was observed that there was a high prevalence of hyperextension and onycholysis of the great toe in patients with HV. A neutral position in extension greater than 30 degrees and the height of the first toe are predisposing factors.

These data have important clinical implications for orthopaedic or surgical interventions in patients with hallux valgus. Treatment of the EHL tendon appears to be crucial for preventing recurrence of onycholysis or progression of HV. Otherwise, abnormal force vectors could persist and counteract positive surgical outcomes. Future studies should therefore focus on interventions targeting the EHL, such as tendon lengthening or reattachment. However, further research is needed to fully understand the underlying biomechanical factors involved.

## Data Availability

The datasets used and/or analysed during the current study are available from the corresponding author upon reasonable request.

## Abbreviations

HV: Hallux valgus
MTP: Metatarsophalangeal
FHL: Flexor hallucis longus
EHL: Extensor hallucis longus
EHB: Extensor hallux brevis
EDL: Extensor digitorum longus
